# PCR effects of melting temperature adjustment of individual primers in degenerate primer pools

**DOI:** 10.7717/peerj.6570

**Published:** 2019-03-04

**Authors:** Ankur Naqib, Trisha Jeon, Kevin Kunstman, Weihua Wang, Yiding Shen, Dagmar Sweeney, Marieta Hyde, Stefan J. Green

**Affiliations:** Sequencing Core, Research Resources Center, University of Illinois at Chicago, Chicago, IL, United States of America

**Keywords:** Microbiome, phiX, Degenerate primers, NGS, Primer design, 16S rRNA

## Abstract

Deep sequencing of small subunit ribosomal RNA (SSU rRNA) gene amplicons continues to be the most common approach for characterization of complex microbial communities. PCR amplifications of conserved regions of SSU rRNA genes often employ degenerate pools of primers to enable targeting of a broad spectrum of organisms. One little noticed feature of such degenerate primer sets is the potential for a wide range of melting temperatures between the primer variants. The melting temperature variation of primers in a degenerate pool could lead to variable amplification efficiencies and PCR bias. Thus, we sought to adjust the melting temperature of each primer variant individually. Individual primer modifications were used to reduce theoretical melting temperature variation between primers, as well as to introduce inter-cluster nucleotide diversity during Illumina sequencing of primer regions. We demonstrate here the suitability of such primers for microbial community analysis. However, no substantial differences in microbial community structure were revealed when using primers with adjusted melting temperatures, though the optimal annealing temperature decreased.

## Introduction

In molecular surveys performed in the field of molecular microbial ecology, genes such as those encoding for the small subunit ribosomal RNA (SSU rRNA), dissimilatory sulfite reductase, nitrite reductase, and more are frequently targeted to survey the total microbial community or members of specific metabolic groups. For broad surveys of microbial community structure, the SSU rRNA gene is most frequently targeted. One common feature of primers used for microbial community surveys is sequence degeneracy; that is, rather than a single primer, a mixture of multiple highly similar primers, targeting multiple variants of priming regions, is employed to cover as broad a taxonomic range of organisms as possible. Over time, the degeneracy of commonly used primers tends to increase due to the availability of additional sequence data demonstrating mismatches between primers and novel sequences. In many cases, introduction of new variants has been highly successful, allowing the detection of specific microbial clades. For example, the commonly used 16S rRNA gene primer 27F does not properly amplify genomic DNA from bacteria of the genus *Bifidobacterium* and other taxa (Hayashi, Sakamoto & Benno, 2004; Frank et al., 2008). A series of additional degeneracies were introduced to improve taxonomic coverage (Frank et al., 2008). More recently, additional degeneracies were added to the 515F primer to allow targeting of *Crenarchaeota* and *Thaumarchaeota* and to 806R to allow targeting of the SAR11 clade (Parada, Needham & Fuhrman, 2016; Apprill et al., 2015).

As new variants are introduced into primer pools, primer melting temperature is rarely considered. Thus, the melting temperature (Tm) of primer variants within a degenerate pool can have a substantial range. For example, the original ‘806R’ degenerate primer employed by the Earth Microbiome Project (EMP) is 18-fold degenerate, with a theoretical Tm range of approximately 7 °C. We previously demonstrated that the annealing temperature of PCRs altered the profile of primers in a degenerate pool annealing to genomic DNA (so-called “primer utilization profiles” or PUP; Green, Venkatramanan & Naqib, 2015). The variable melting temperature of the individual primers in a degenerate pool creates the potential for additional bias, as differences in free energy binding between primers and perfectly matching templates will vary. Furthermore, mismatch interactions could be favored when primers with high theoretical Tm values are used in PCR reactions run at low annealing temperatures necessary for low Tm primers. Alternatively, some primers may not have the opportunity to anneal in PCR reactions with elevated annealing temperatures. Such reactions can still yield amplification due to the presence of high Tm primers in the degenerate pool.

The objective of this study was to develop and test an experimental system to determine whether the broad range of primer melting temperatures in degenerate primer pools contributes substantially to observed microbial community profiles generated from amplicon-based sequencing approaches. To do so, we altered each primer independently by removing nucleotides from both 5′ and 3′ ends of oligonucleotide primers to minimize variance in primer theoretical Tm. These oligonucleotides were synthesized independently and pooled in equimolar concentration. Subsequently, these primer pools were used for PCR amplification of several complex genomic DNA samples, followed by high-throughput sequencing. Sequencing results were compared to amplicon sequence data generated from PCRs employing standard degenerate primers. As a secondary objective, we sought to determine if we could modify primer sequences at the 5′ end to introduce nucleotide diversity into amplicons sequenced on next-generation sequencers.

## Materials and Methods

### Primer design and synthesis

The most recent Earth Microbiome Project (EMP) primers, 515F (Parada) [“515F”] and 806R (Apprill) [“806R”], were utilized as the default primer set (Parada, Needham & Fuhrman, 2016; Apprill et al., 2015; Walters et al., 2016; Quince et al., 2011). These primers are 4-fold degenerate (515F) and 24-fold degenerate (806R), with theoretical melting temperatures ranging from 66.9 to 71.8 °C (515F) and from 54.7 to 61.7 °C (806R) (Table S1). Primer theoretical melting temperatures were calculated using the OligoAnalyzer3.1 calculator (Owczarzy et al., 2008), assuming 250 nM primer concentration, 2 mM Mg^2+^, and 0.2 mM dNTPs. Maximum Delta G values for each sequence were calculated using the self-dimer option in the OligoAnalyzer software. Primers were synthesized either as single degenerate primer pools (standard approach), or as individual primers without degeneracies by Integrated DNA Technologies (IDT, Coralville, IA, USA). All primers were synthesized as LabReady and delivered at a fixed concentration of 100 micromolar. Most primers contained common sequence linkers (CS1 and CS2) at the 5′ ends, as shown in Table S2. These linker sequences are necessary for the later incorporation of Illumina sequencing adapters and sample-specific barcodes. For new primer pools containing shortened primers, a combination of either four (“515F”) or 24 (“806R”) non-degenerate primers were combined in equal volume to generate degenerate pools at 100 micromolar concentration. These primer pools were named “515F_pool” and “806R_pool”, and when these primers were used together, the primer set was named “ShortEMP”. One experiment, described below, employed ShortEMP primer pools without common sequences (“NoLinker_ShortEMP”) to assess the effect of the linker sequences on analysis of microbial community structure. A final set of primers, employing variable length spacers, was generated and named the “LongEMP” primer set. These primers included variable length spacers between the common sequence linkers and the gene-specific EMP primer regions, as described previously (Lundberg et al., 2013). Sequencing with these primers was performed on both MiniSeq and MiSeq instruments, but data from the MiniSeq using the “LongEMP” primer set did not properly merge due to read-length limitations, and was not further analyzed.

### Genomic DNA templates

Four microbial genomic DNA (gDNA) samples were employed in this study. These include the ZymoBIOMICS Microbial Community DNA standard (D6306; Zymo Research, Irvine, CA, USA; ‘Zymo’), as well as three environmental samples derived from Lake Michigan sediment (‘LMC’), garden soil (‘Soil’) and mammalian (rat) feces (‘Feces’). Sediment, soil and fecal samples were extracted using a PowerSoil DNA extraction kit (Qiagen, Hilden, Germany), following the manufacturer’s protocol.

### Amplicon library preparation and sequencing

A two-stage PCR amplification strategy was used to generate sequencer-ready amplicons (Naqib et al., 2018). Genomic DNA was first PCR amplified with primer set EMP (CS1_806R and CS2_515F), ShortEMP (CS1_806R_pool and CS2_515F_pool), or LongEMP (CS1_806R_long_pool and CS2_806R_long_pool) (Table S2). All primers contained 5′ common sequence tags (known as common sequence 1 and 2, CS1 and CS2) as described previously (Moonsamy et al., 2013). First stage PCR amplifications were performed in 10 microliter reactions in 96-well plates, using MyTaq HS 2X mastermix (Bioline, Taunton, MA, USA). PCR conditions were 95 °C for 5 min, followed by 28 cycles of 95 °C for 30″, variable annealing temperature for 60″and 72 °C for 90″. For temperature gradients, annealing temperatures of 40°, 45° and 50 °C were employed. Subsequently, annealing temperatures of 45 °C were used for ShortEMP reactions and 50 °C for EMP reactions.

Second stage PCR amplifications were performed in 10 microliter reactions in 96-well plates. A mastermix for the entire plate was made using the MyTaq HS 2X mastermix. Each well received a separate primer pair with a unique 10-base barcode, obtained from the Access Array Barcode Library for Illumina (Fluidigm, South San Francisco, CA; Item# 100-4876). These AccessArray primers contained the CS1 and CS2 linkers at the 3′ ends of the oligonucleotides. Cycling conditions were as follows: 95 °C for 5 min, followed by 8 cycles of 95 °C for 30″, 60 °C for 30″and 72 °C for 30″.

In an experiment using the NoLinker_ShortEMP primers, the above protocol was modified slightly. To assess the effect of common sequence linkers on the observed microbial community structure, we performed the first stage PCR amplification with NoLinker_ShortEMP primers. PCR conditions were identical to those described above. During the second stage PCR amplification, both Fluidigm Access Array Barcode and the ShortEMP primers were included, and 12 cycles of amplification were performed in place of eight. In this approach, common sequences are incorporated during the second stage PCR as the ShortEMP primers *with* linkers amplify the NoLinker_ShortEMP amplicons; subsequently, the Fluidigm Access Array barcode primers amplify amplicons containing the common sequence linkers.

In all experiments, samples were pooled using an EpMotion5075 liquid handling robot (Eppendorf, Hamburg, Germany). Pooled libraries were purified using an AMPure XP cleanup protocol (0.6X, vol/vol; Agencourt, Beckmann-Coulter) to remove fragments smaller than 300 bp. The pooled libraries, with either a 1% or 20% phiX spike-in, were loaded onto either an Illumina MiniSeq mid-output kit (2 × 153 paired-end reads) or an Illumina MiSeq V2 kit (2 × 250 paired-end reads), as indicated in Table S3. Fluidigm sequencing primers, targeting the CS1 and CS2 linker regions, were used to initiate sequencing. De-multiplexing of reads was performed on instrument. Library preparation, pooling, and sequencing were performed at the University of Illinois at Chicago Sequencing Core (UICSQC).

### Bioinformatic analysis of sequence data

Raw FASTQ files were downloaded from Illumina Basespace. Sequence reads were merged using PEAR (Paired-End Read Merger) (Zhang et al., 2013) with default parameters. Merged reads were quality trimmed (<Q20 discarded) and length trimmed (<250 bases were removed), and primer sequences were removed using the ‘Trim reads’ algorithm within the software package CLC Genomics Workbench (v11; Qiagen; Hilden, Germany). Trimmed sequences were reverse complemented using a QIIME script. Chimeras were removed using the USEARCH81 algorithm (Edgar, 2010). Subsequently, for environmental samples (LMC, Soil, Feces), sequences were pooled, renamed and clustered into operational taxonomic units (OTUs) at a threshold of 97% similarity (QIIME v1.8.0; Caporaso et al., 2010). Each OTU was annotated taxonomically based on the representative sequences using the UCLUST algorithm and the greengenes 13_8 reference database (McDonald et al., 2012b). A biological observational matrix (BIOM) was generated from the clustered OTU data and the taxonomy data (McDonald et al., 2012a). For subsequent analyses, data were rarefied using the *vegan* package within the R programming language. Rarefaction depths were adjusted by analysis; depth of rarefaction for each figure is shown in Table S3. The BIOMs were analyzed and visualized using the software package Primer7 (Clarke, 1993) and in the R programming environment (R Core Team, 2013). Dendrogram creation and SIMPROF tests were conducted within Primer7. The *vegan* package (Oksanen et al., 2011) was used to generate alpha diversity indices and to calculate pairwise Bray–Curtis dissimilarity scores. Analysis of similarity (ANOSIM) calculations were performed at the taxonomic level of genus, using square root transformed data. Metric multi-dimensional scaling (mMDS) plots were created using the cmdscale and ggplot2 functions within R. Ellipses, representing a 95% confidence interval around group centroids, were created assuming a multivariate *t*-distribution (Fox & Weisberg, 2011). Taxon-level differential abundances between sample groups were identified using the software package STAMP  (Parks et al., 2014) employing White’s non-parametric *t*-test (White, Nagarajan & Pop, 2009). *P* values were adjusted using the Benjamini–Hochberg False Discovery Rate correction  (Hochberg & Benjamini, 1990).

Sequence data from ‘Zymo’ samples were processed using the same pipeline, but data were not clustered. Instead, merged, trimmed and chimera-cleaned data were mapped against reference gene sequences for the eight bacterial reference organisms using the software package CLC genomics workbench v10 (Qiagen, Aarhus, Denmark). The eight reference organisms included: *Lactobacillus fermentum* (AJ575812), *Bacillus subtilis* (DQ993674), *Escherichia coli* (J01859), *Enterococcus faecalis* (EU887827), *Salmonella enterica* (JQ694167), *Listeria monocytogenes* (M58822), *Pseudomonas aeruginosa* (LN874213), and *Staphylococcus aureus* (L37597). Mapping data were then converted to biological observation matrices for use in visualization and statistical analyses, as described above. Ideal scores were calculated according to the formula described previously (Green, Venkatramanan & Naqib, 2015). Briefly, the Ideal score is a summation of the absolute difference between the expected relative abundance and the observed relative abundance for each feature in a multi-feature dataset.

### Data sharing

Raw sequence data files were submitted in the Sequence Read Archive (SRA) of the National Center for Biotechnology Information (NCBI). The BioProject identifier for the samples is PRJNA492144. Full metadata for each sample are provided in Table S3.

## Results

### Primer design

**Figure 1:**
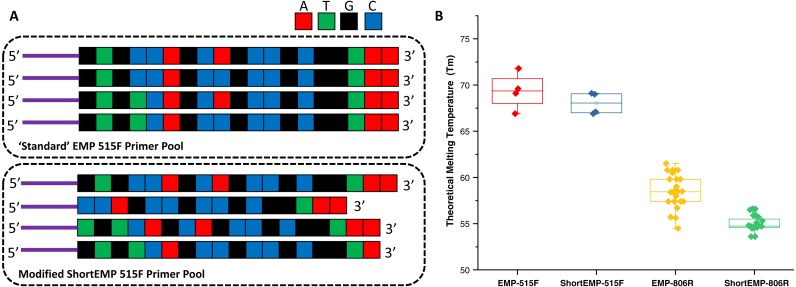
Schematic of primer design and primer theoretical melting temperature distribution. (A) Standard degenerate primer pools are synchronized and have low nucleotide diversity when sequenced on Illumina sequencers. Nucleotides were removed from the 5′ ends of locus-specific portions of oligonucleotide primers to adjust melting temperature and to introduce nucleotide diversity. Nucleotides were removed from the 3′ ends of locus-specific portions of primers to adjust melting temperature only. Sequencing reactions are initiated using the ‘common sequences’ (purple lines) adjacent to the locus-specific regions of the PCR primers. (B) Distribution of theoretical melting temperatures (Tm, °C) for primer pools using standard EMP primers and modified ShortEMP primers. Modified 515F primer pool Tm distribution is not significantly different from the standard 515F primer pool (Wilcoxon-Mann-Whitney test; *p* = 0.37), while modified 806R primer pool Tm distribution is significantly different from the standard 806R primer pool (WMW test; *p* < 0.00001).

The design of new primers with lower variation in melting temperature was performed with two constraints, including: (a) no primer shorter than 16 bases, and (b) all degenerate positions are retained. Outside of these constraints, we aimed to minimize theoretical melting temperature variation within each degenerate primer pool. The high overall GC content and 3′ run of A or T bases in the 515F primer limited our ability to adjust Tm of this primer while meeting the above constraints. An underlying assumption of the primer modification was that shortening the primers could only increase the range of potential targets (Isenbarger et al., 2008). To adjust primer Tm, bases were sequentially removed from either or both 5′ and 3′ ends of each oligonucleotide (Fig. 1A; Table S1). The final primer design (“ShortEMP”) yielded pools of primers with overall lower average theoretical melting temperatures, and with smaller range in Tm between all primers within the pool compared to the original pool (“EMP”) (Fig. 1B; Table S1). The 515F primer pool, comprised of four primers, was only modestly affected by the primer alterations. The Tm range prior to modification was 66.9 to 71.8 °C; after modification, the Tm range was 66.9 to 69.1 °C. The Tm range of the modified primer pool was not significantly different from that of the original pool [Wilcoxon–Mann–Whitney (WMW) test; *p* = 0.37]. The 806R primer pool, comprised of 24 primers was more strongly affected by the alterations. The Tm range prior to modification was 54.7 to 61.7 °C; after modification, the Tm range was 53.6 to 56.6 °C. The Tm range of the modified primer pool was significantly different from that of the original pool (WMW test; *p* < 0.00001).

A secondary aim of design was to introduce nucleotide diversity at the 5′ end of the combined oligonucleotides by preferential removal of bases as the 5′ end of the gene-specific portion of each primer (Fig. 1A; Tables S1, S4 and S5). Illumina’s technical notes indicate that having relatively even proportions of all four nucleotides during each sequencing cycle is necessary for proper sequencing. This is particularly true during the first cycles of sequencing, when the sequencer is still training itself to identify clusters. To improve sequence quality of so-called “low diversity” libraries (e.g., 16S rRNA gene amplicon libraries), exogenous spike-ins of shotgun DNA libraries derived from the virus phiX174 are typically employed. Elsewhere, Lundberg et al. (2013) introduced nucleotide diversity through the synthesis of primers containing frameshift nucleotides. Briefly, a mixture of six different forward and six different reverse primers were pooled together (Lundberg et al., 2013). Each primer variant contained an identical gene-specific degenerate primer at the 3′ end of the oligonucleotides, but a variable number of nucleotides upstream of the gene-specific portion of the primers. This variable number of upstream nucleotides introduces artificial diversity into the sequencing reaction due to an effective frame shift by offsetting the start of highly conserved regions of the amplicon by 1–5 bases. We aimed to achieve the same effect by removing bases from the 5′ end of the gene-specific portion of the primers, thereby introducing the frame-shift effect (Fig. 1A). To assess the effect of 5′ nucleotide removals on the overall nucleotide diversity at each position, we generated two *in silico* calculations: (a) number of different nucleotides present at any given position across the first 16 nucleotides of each primer pool, and (b) the Shannon index for nucleotide diversity at each of the first 16 nucleotides of each primer pool (Tables S4 and S5). The original primer pool design has very low nucleotide diversity, as the primers are perfectly synchronized and have only two or three degenerate positions. Thus, for the original 515F primer pool, all primer positions other than the 4th and 9th positions have only a single nucleotide represented. The average number of nucleotides present at any position is 1.125, and the average Shannon index (natural log) across the first 16 positions is 0.09 (Table S4). Conversely, after modifying the individual primers, the average nucleotide diversity across the 16 positions was 2.44, with an average Shannon index of 0.77. The maximum possible Shannon index value for 4 different nucleotides is 1.39. A similar effect was observed for the 806R primer pool (Table S5). Prior to redesign, the average nucleotide diversity across the first 16 positions was 1.38, compared to 3.19 after redesign. Likewise, Shannon index increased from 0.2 to 1.0 after redesign.

### Microbiome profiling using EMP and ShortEMP primers

**Figure 2:**
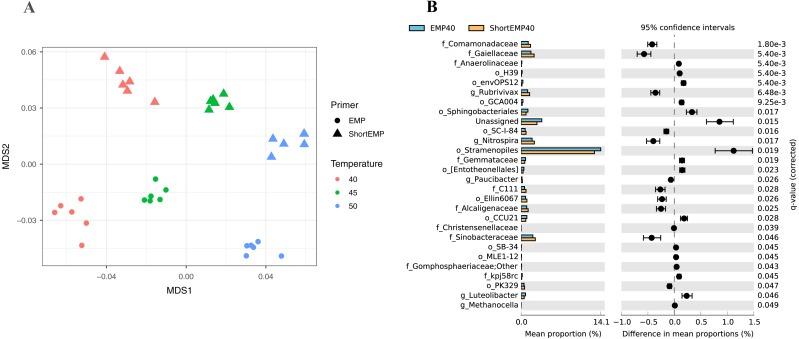
Effect of annealing temperature and primer set on observed microbial community in Lake Michigan sediment. A composite sample of gDNA from Lake Michigan sediment was amplified using EMP or ShortEMP primer pools at annealing temperatures of 40, 45 and 50 °C and sequenced on an Illumina MiniSeq instrument. Sequence data were rarefied to a depth of 17,500 sequences per sample. (A) Genus-level annotations of sequence data were visualized using metric multidimensional scaling (mMDS) employing a distance matrix based on Bray–Curtis similarity. Across all temperatures, the use of EMP and ShortEMP primers resulted in slightly, but significantly, different observed microbial communities (ANOSIM *R* = 0.473; *p* < 0.001; MDS axis 2). Increasing annealing temperature also led to significant changes in observed microbial community structure (ANOSIM *R* = 0.694, *p* < 0.001; MDS axis 1). Observed microbial community structures at all temperature and primer set combinations were significantly different (ANOSIM R values 0.748–1.0; *p* < 0.002). (B) A taxon-by-taxon analysis was performed to identify taxa with significantly different relative abundance between EMP and ShortEMP primer sets. Shown is the comparison between EMP and ShortEMP primers at 40 °C annealing temperature; comparisons at 45 °C and 50 °C are shown in Fig. S3. Genus-level annotations are shown (when available), and the mean relative abundance (six technical replicates) for each primer set is shown, together with the difference in mean proportions. For each comparison a *q*-value, calculated in the software package STAMP using White’s non-parametric *t*-test along with a Benjamini-Hochberg FDR correction, is shown. Only significantly differently abundant taxa (*q* < 0.05) are shown. Sequences annotated as Stramenopiles are derived from SSU rRNA genes of chloroplasts from these organisms.

**Figure 3:**
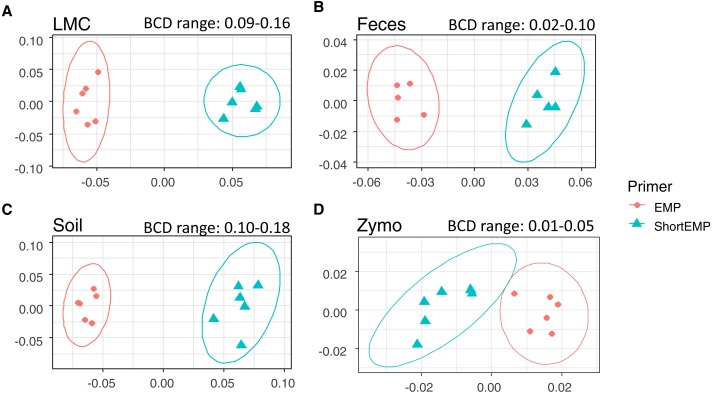
Effect of primer set on observed microbial communities in complex microbial samples. Genus-level annotations of sequence data sequenced on an Illumina MiSeq instrument were visualized using mMDS ordination employing a distance matrix based on Bray–Curtis similarity. *X*-axes represent MDS axis 1 and *y*-axes represent MDS axis 2 for all sample types. Sequence data were rarefied to different depths for each sample type (Table S3). For each sample, six technical replicates were performed at optimal annealing temperatures of 45 °C (ShortEMP) and 50 °C (EMP). Small, but significant, shifts in microbial communities were observed between EMP and ShortEMP primers for (A) Lake Michigan sediment (LMC), ANOSIM *R* = 1, *P* = 0.0021, (B) rat feces (Feces), ANOSIM *R* = 1, *P* = 0.0027, (C) garden soil (Soil), ANOSIM *R* = 1, *P* = 0.0033, and ZymoBIOMICS Microbial Community DNA standard (Zymo), ANOSIM *R* = 1, *P* = 0.0019. The range of Bray–Curtis dissimilarity (BCD) between EMP and ShortEMP technical replicates is shown above each figure. Ellipses represent a 95% confidence interval around the centroid. Two outliers from the fecal analysis were removed (see Fig. S2).

To assess the effects of primer modifications, initially a single complex genomic DNA template (LMC) was profiled across an annealing temperature gradient using EMP and ShortEMP primer sets. We previously observed a strong and consistent shift in microbial community structure associated with increasing annealing temperatures from 40 °C to 55 °C, with specific taxa such as Prevotellaceae increasing in relative abundance with increasing annealing temperature in mammalian fecal samples (Green, Venkatramanan & Naqib, 2015). Therefore, annealing temperatures of 40 °C, 45 °C and 50 °C were tested in this study. The single gDNA template was PCR amplified at each of the temperatures and primer sets with six technical replicates. The observed microbial community from the source gDNA template was significantly affected by primer set (EMP or ShortEMP) and by annealing temperature (Fig. 2). Variation in microbial community structure by temperature was described primarily by the MDS axis 1, while that of primer set was described primarily by MDS axis 2. The magnitude of the shift was small, with overall Bray–Curtis similarity of all comparisons between EMP and ShortEMP replicates >0.84 (Fig. 3A). Within each primer set, replicates had Bray–Curtis similarity values >0.89, with ShortEMP replicates having slightly and significantly greater similarity (Fig. S1, LMC). No significant effect of primer set and annealing temperature on alpha diversity indices (e.g., richness and Shannon index) at the genus- or OTU-level were observed for LMC (Table 1). At each annealing temperature, relatively few taxa (16–28 genus-level taxa) were significantly differently abundant between the EMP and ShortEMP analyses (Fig. 2B, Fig. S3).

**Table 1:** Alpha diversity indices of observed microbial communities. Shannon indices and richness were calculated at the taxonomic levels of genus and at the operational taxonomic unit (OTU97) level. For each primer set (EMP or ShortEMP), an average and standard deviation of six technical replicates is shown. Kruskal-Wallis tests (KW) were performed to determine if observed diversity was significantly different between EMP and ShortEMP analyses, and *p*-values are shown. Diversity indices were not significantly different between primer sets with the exception of OTU-level Shannon index for the Soil sample.

**Sample**	**OTU shannon**	**OTU richness**	**Sample**	**Genus shannon**	**Genus richness**
EMP_LMC_40	6.88 ± 0.05	3787.60 ± 97.70	EMP_LMC_40	4.76 ± 0.02	545.60 ± 11.35
EMP_LMC_45	6.81 ± 0.09	3738.80 ± 81.14	EMP_LMC_45	4.74 ± 0.06	546.00 ± 13.21
EMP_LMC_50	6.86 ± 0.02	3810.80 ± 57.22	EMP_LMC_50	4.77 ± 0.02	545.00 ± 10.89
ShortEMP_LMC_40	6.81 ± 0.10	3708.60 ± 77.73	ShortEMP_LMC_40	4.76 ± 0.04	534.60 ± 10.78
ShortEMP_LMC_45	6.82 ± 0.02	3780.00 ± 42.27	ShortEMP_LMC_45	4.73 ± 0.01	533.00 ± 4.18
ShortEMP_LMC_50	6.86 ± 0.06	3862.80 ± 107.70	ShortEMP_LMC_50	4.77 ± 0.03	542.20 ± 5.81
	*p* = 0.134	*p* = 0.139		*p* = 0.107	*p* = 0.161
EMP_Soil_50	7.16 ± 0.03	2907.50 ± 99.02	EMP_Soil_50	4.82 ± 0.02	414.50 ± 10.45
ShortEMP_Soil_45	7.09 ± 0.04	2832.67 ± 65.56	ShortEMP_Soil_45	4.80 ± 0.03	418.17 ± 24.17
	***p***=**0.010**	*p* = 0.200		*p* = 0.262	*p* = 0.873
EMP_Feces_50	4.94 ± 0.04	563.17 ± 25.34	EMP_Feces_50	2.41 ± 0.00	50.00 ± 2.37
ShortEMP_Feces_45	4.90 ± 0.08	605.17 ± 65.49	ShortEMP_Feces_45	2.39 ± 0.05	52.50 ± 2.51
	*p* = 0.200	*p* = 0.055		*p* = 0.055	*p* = 0.089

Based on the results of the temperature gradient analysis (above) and testing of a mock community standard (below), an annealing temperature of 45 °C was chosen for additional analyses employing the ShortEMP primers and 50 °C for EMP primers. Two additional complex microbial samples were analyzed at these temperatures, with six technical replicates for each. These samples include ‘feces’ and ‘soil’. In each of these analyses, a significant effect of primer set was observed (Figs. 3B and 3C; ANOSIM *R* = 1, *p* < 0.008), and in each case, the magnitude of the effect was similar, with overall Bray–Curtis similarity between all replicates of the same sample >0.82 (Figs. 3B and 3C). Small effects of primer set on alpha diversity indices were observed, and only the OTU-level Shannon index for the soil sample was significantly different between ShortEMP primers relative to EMP primers (7.09 vs 7.16) (Table 1).

### Interrogation of a mock community with EMP, ShortEMP, LongEMP and NoLinker_ShortEMP primer sets

The ZymoBIOMICS Microbial Community DNA standard (“Zymo”) was used as a gDNA template to assess the capability of the various primer sets to characterize microbial communities. The Zymo standard is composed of 8 bacterial taxa and 2 fungal taxa at varying levels of abundance (fungi are not amplified by the 515F/806R primer set). When analyzed using 16S rRNA gene amplicon sequencing, the relative abundance of each strain should range from 4.2% to 18.4%. Both primer sets (EMP and ShortEMP) generated highly similar but significantly distinct results (Fig. 3D; ANOSIM *R* = 1, *p* = 0.0019). For each replicate, an Ideal Score (Green, Venkatramanan & Naqib, 2015) was calculated based on the expected relative abundance of each taxon. The Ideal Score represents a summation of the difference in relative abundance of each taxon in a mock community. A perfect representation of the expected relative abundance of each taxon will yield an ideal score of zero, with values greater than zero representing increasing discordance from the expected results. Ideal score results were generated for each of the six technical replicates and compared at annealing temperatures of 40 °C, 45 °C and 50 °C for EMP and ShortEMP primers (Table 2). When employing the EMP primers for analysis of the Zymo standard, annealing temperatures below 50 °C produced progressively worse results (i.e., higher ideal scores). When employing the ShortEMP primers for analysis of the Zymo standard, annealing temperatures of 45 °C and 50 °C yielded similar results, with low ideal scores relative to 40 °C (Table 2).

**Table 2:** Ideal score calculations for analysis of the ‘Zymo’ Standard. The Ideal score is a summation of the absolute difference between the expected relative abundance and the observed relative abundance for each feature in a multi-feature dataset. Lower Ideal scores indicate a better representation of the expected community. Kruskal-Wallis test (KW) *p*-values are shown for comparisons across temperatures or between EMP and ShortEMP primers at single temperatures.

**Annealing Temp.**	**EMP (SD)**	**ShortEMP (SD)**	**KW**
**40 °C**	30.33 (1.75)	25.10 (6.29)	*p* = 0.054
**45 °C**	16.03 (1.54)	12.95 (0.98)	*p* = 0.006
**50 °C**	8.95 (1.52)	13.33 (1.49)	*p* = 0.006
	*p* = 0.001	*p* = 0.003	
	EMP50 (SD)	ShortEMP45 (SD)	
**ShortEMP 45 °C vs EMP 50 °C**	8.95 (1.52)	12.95 (0.98)	*p* = 0.006

**Notes.**

SD standard deviation

Ideal scores indicated that when used at their optimal annealing temperature EMP primers were slightly, but significantly, better than ShortEMP primers in recovering the expected distribution of the Zymo bacterial taxa. Although the EMP primers slightly outperformed the ShortEMP when compared to the expected distribution of eight bacterial taxa, the ShortEMP primers better tolerated a broader range of annealing temperatures. ShortEMP primers produced nearly identical results at 45 °C and 50 °C (Table 2), demonstrating that the lower overall Tm of the primer pool can contribute to a shift in optimum annealing temperature.

**Figure 4:**
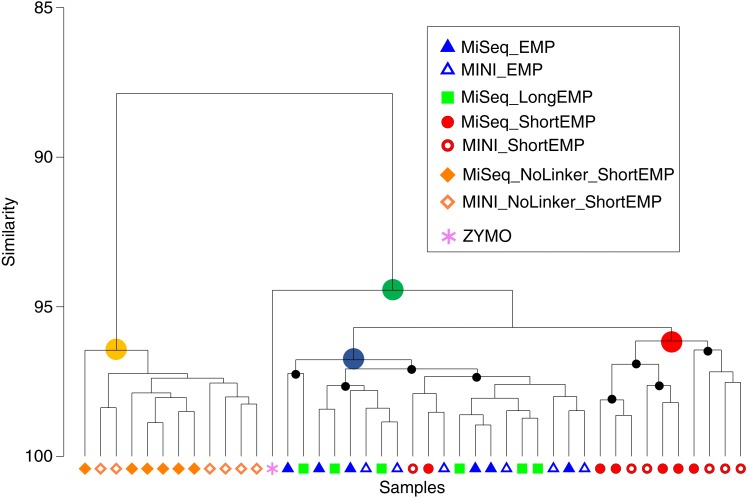
Comparison of representation of a mock community standard using all primer sets and sequencing platforms. A single mock community standard (‘Zymo’) consisting of 8 bacterial taxa was amplified using EMP, ShortEMP, LongEMP and NoLinker_ShortEMP primers using both MiniSeq and MiSeq platforms and a dendrogram representing Bray–Curtis similarity is shown. Rarefaction was performed to a depth of 4,000 sequences per sample. The similarity profile routine (SIMPROF) test was performed to identify clusters with non-random structure. Nodes with a non-random structure (significance level of 5%) are indicated with black or colored circles. The ideal representation of the standard is shown as a pink asterisk. The observed community generated using NoLinker_ShortEMP primers (node with yellow circle) was significantly divergent from those observed with all other primer sets (ANOSIM *R* = 1, *p* = 0.002) and from the Zymo standard, and a significant effect of sequencing platform was observed with these primers (*R* = 0.416, *p* = 0.002, 462 permutations) The microbial communities observed with EMP and LongEMP primers were most similar to each other (node with blue circle). No significant effect of sequencing platform was observed with EMP primers (*R* = 0.166, *p* = 0.091) or ShortEMP primers (node with red circle; ANOSIM *R* = 0.05, *p* = 0.281). The microbial communities observed with EMP, LongEMP and ShortEMP clustered together with the Zymo standard (node with green circle). No data are shown for LongEMP with the MiniSeq platform, due to incomplete merging of paired-end reads.

We sought to assess whether the addition of spacer regions, as described previously by Lundberg et al. (2013), would alter the efficiency of amplification in this system. We synthesized primers containing common sequence linkers, a 0–5 base frameshift sequence, a two-base ‘linker’, and the EMP primer sequence (Table S2; ‘LongEMP’). A pool of six forward primers and six reverse primers were used in the standard two-stage PCR protocol to amplify the mock community gDNA. The observed mock community generated from LongEMP analyses was most similar, and not significantly different, from that generated using the EMP primer set (Fig. 4; ANOSIM *R* =  − 0.179, *p* = 0.965). EMP and LongEMP primer sets generated more similar observed microbial communities, relative to the ShortEMP primer set (ANOSIM *R* = 0.715 − 0.722, *p* = 0.002). Although significant, the difference was not large, and EMP, ShortEMP and LongEMP generated highly similar results when applied to the mock community DNA standard (Fig. 4).

We also sought to assess whether the common sequencer linkers themselves contributed to distortion of the underlying microbial community structure. Conceptually, the 3′ ends of the *linker* sequences could interact with genomic DNA templates, leading to preferential amplification of some templates. To avoid initial interaction of linker sequences with gDNA templates, we performed the first stage PCR amplification with ShortEMP primers *without* common sequence linkers (NoLinker_ShortEMP). After 28 cycles of amplification, the generated PCR amplicons were transferred to the second stage PCR amplification, as performed for all other reactions. To allow these amplicons without linkers to be prepared for Illumina sequencing, both ShortEMP primers (with common sequence linkers) and Fluidigm Access Array barcoding primers were added to the second stage reaction. Final amplicons were sequenced and the resulting sequence data analyzed together with EMP, ShortEMP and LongEMP primer sets (Fig. 4). The observed community generated by the NoLinker_ShortEMP primer set was significantly different than those generated by EMP, ShortEMP and LongEMP primer sets (ANOSIM *R* = 1, *p* = 0.002), and the observed community structure was also more divergent from the expected structure than those generated with the other primer sets (Fig. 4). Thus, the common sequence linkers do not appear to substantially alter the observed mock microbial community structure, and removing the linkers leads to a more complex workflow and a poorer representation of the mock community.

We should note that the Zymo standard is not an ideal mock community for assessing the action of a degenerate primer pool. We sought to identify the sequences of the eight bacterial DNA templates in the region of the 515F and 806R primers, and despite the moderately broad range of bacterial taxa included (Proteobacteria, Actinobacteria, and Firmicutes), all sequences were identical at the primer annealing locations. This mock community is, therefore, appropriate for determining that an overall PCR and sequencing workflow is successful but is not a good approximation of a highly complex natural microbial community, where even conserved sites such as the 515F and 806R primer sites contain considerable sequence heterogeneity.

We also used the amplicons generated from the Zymo standard to assess variability introduced by sequencing on the Illumina MiSeq or MiniSeq platforms. For the EMP, ShortEMP, and NoLinker_ShortEMP, identical amplicons were sequenced and analyzed on both platforms. The LongEMP amplicon reads generated on the Illumina MiniSeq did not consistently merge, and were not included in the analysis. This is due to the additional ‘spacer’ bases (up to six on each read) and the short overlap of paired-end reads generated with 2 × 153 bases reads on the MiniSeq. MiSeq and MiniSeq results from the same amplicons clustered together, regardless of primer set (Fig. 4). For the EMP and ShortEMP primer sets, the effect of sequencing platform was not significant (ANOSIM *R* = 0.166, *p* = 0.091 and *R* = 0.05, *p* = 0.281, respectively). For the NoLinker_ShortEMP primer set, a significant effect of sequencing platform was observed (ANOSIM *R* = 0.416, *p* = 0.002). Thus, a slight effect of sequencing platform was observed, but the choice of platform did not alter biological conclusions.

### Assessing the need for phiX spike-in with ShortEMP primers

**Figure 5:**
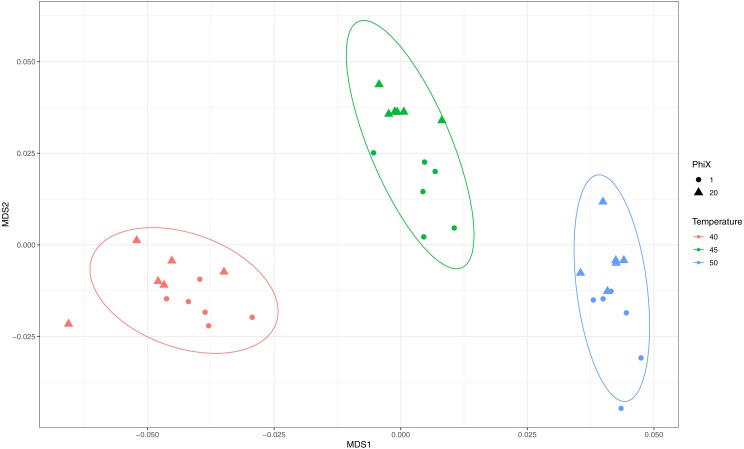
Effect of phiX spike-in on observed microbial communities with amplicons generated using ShortEMP primers. Genus-level annotations of sequence data generated on an Illumina MiniSeq instrument were visualized using mMDS ordination employing a distance matrix based on Bray–Curtis similarity. Rarefaction was performed to a depth of 30,000 sequences per sample. For each ShortEMP PCR condition, six technical replicates were analyzed using either 20% or 1.75% phiX spike-in. Small differences, consistent with run-to-run variation, were observed between the two sequencing runs (ANOSIM: 40 °C *R* = 0.146, *p* = 0.052; 45 °C *R* = 0.394, *p* = 0.011; 50 °C *R* = 0.178, *p* = 0.100). Ellipses represent a 95% confidence interval around the centroid.

We sequenced ShortEMP amplicons on an Illumina MiniSeq run without substantial phiX spike-in to determine if ShortEMP amplicons by themselves produced sufficient nucleotide diversity to allow proper clustering. These amplicons, generated from Lake Michigan sediment, at annealing temperatures of 40, 45 and 50, were analyzed together with the same amplicons generated on a MiniSeq run with a 20% phiX spike-in. The overall quality of the run was extremely high (>96% pass-filter, >97% Q30 with approximately 1.75% phiX spike-in), and results were similar to those generated on a 20% phiX run (Fig. 5). Slight trends towards small, significant differences were observed between the two sequencing runs, but the overall magnitude of the difference was small.

## Discussion

We demonstrate here that the strategy of removing nucleotides from the 5′ ends of individual primers is effective for introducing nucleotide diversity into a primer pool, and for reducing the Tm range of the primers within a degenerate primer pool. Selective removal of bases at the 3′ end can be used together with 5′ base removal to adjust overall primer melting temperature. Base removal at the 3′ end, when using the Fluidigm sequencing protocol, does not impact nucleotide diversity for the purposes of Illumina sequencing. In this study, to ensure that the greatest nucleotide diversity was present during the initial cycles of the first sequencing reaction, the ‘CS1’ linker sequence was attached to the 806R primer. This approach is inverted compared to standard EMP workflows, where the CS1 linker is attached to the 515F primer (Naqib et al., 2018). For future designs, the choice of linker can be adjusted as needed to ensure the highest nucleotide diversity during the initial cycles of the first read of Illumina sequencers, when cluster identification is performed. When amplicons generated with the modified primers were sequenced on an Illumina MiniSeq sequencer with <2% phiX, the data quality and community analyses were consistent with the same amplicons generated on a run with 20% phiX. Therefore, we conclude that the nucleotide diversity created by removing bases at the 5′ ends of primers to effect the frameshift was sufficient to allow for sequencing without substantial phiX inclusion.

We sought to determine whether the broad range of primer melting temperatures in degenerate primer pools contributes substantially to observed microbial community profiles. Individual primer modifications were used to reduce theoretical melting temperature variation between primers within a degenerate pool, and the modified primers were used to amplify mock and environmental samples. When compared to amplicons generated using standard primer sets, the reducing melting temperature variability in the modified primer pools did not substantially alter observed microbial community structure in the tested samples. The modified primers had limited or no effect on measured alpha diversity in complex microbial samples and shifts in microbial community structure associated with the modified primers relative to the standard primers were small in scale. The shortened primers appear to have greater tolerance for lower annealing temperatures than the standard primers. We considered the possibility that high Tm primers could dominate primer-template interactions, leading to variable amplification efficiencies in PCR and reduced taxonomic coverage. Our results, however, do not support any substantial modification in the target range for the modified primers, even at low annealing temperatures. We do note, however, that the 515F and 806R primers themselves have highly divergent theoretical melting temperatures, and that this discrepancy could contribute to the difficulty in expanding the targeted taxonomic range of PCR-based microbiome sequencing.

## Conclusions

We demonstrate here a novel method to introduce nucleotide diversity into PCR amplicons for sequencing on Illumina sequencers. Through selective removal of bases at the 5′ end of oligonucleotide primers, nucleotide diversity can be introduced without substantial effect on the activity of the primers themselves. When employing this strategy with a commonly-used primer set targeting microbial SSU rRNA genes, no substantial effects on observed microbial community structures were observed.

##  Supplemental Information

10.7717/peerj.6570/supp-1 Figure S1 Box plots of within-group Bray–Curtis dissimilarity scores for microbiome analyses conducted with EMP and ShortEMP primer sets For each sample, Bray–Curtis dissimilarity was calculated for 6 technical replicates with EMP primers (15 comparisons), and 6 technical replicates with ShortEMP primers (15 comparisons). A comparison of median within-sample similarity for replicates from EMP and ShortEMP amplifications was performed, and were significantly different for LMC, Feces and Soil (Mann-Whitney test, *P* < 0.053). An outlier of one replicate from both EMP and ShortEMP fecal analyses was removed (see Fig. S2).

10.7717/peerj.6570/supp-2 Figure S2 Analysis of fecal sample replicates including outliers A single technical replicate, representing an outlier, was removed from both EMP and ShortEMP analyses (Fig. 3 and Fig. S1). Analyses in this figure are shown including the deep outliers. Inclusion of the outliers does not modify the conclusions of the analysis. However, the ShortEMP outlier is greatly different from all other technical replicates from all samples in the study. (A) Genus-level annotations of sequence data were visualized using mMDS ordination employing a distance matrix based on Bray–Curtis similarity. Six technical replicates were performed at optimal annealing temperatures of 45 °C (ShortEMP) and 50 °C (EMP). Small, but significant, shifts in microbial communities were observed between EMP and ShortEMP primers for Feces (ANOSIM *R* = 0.68, *P* = 0.0025). Bray–Curtis dissimilarity (BCD) between EMP and ShortEMP technical replicates is shown above the figure. Ellipses represent a 95% confidence interval around the centroid. (B) Box plot of within-group Bray–Curtis dissimilarity scores for microbiome analyses conducted with EMP and ShortEMP primer sets on fecal DNA.

10.7717/peerj.6570/supp-3 Figure S3 Significant differences in taxon relative abundance between EMP and ShortEMP analyses of Lake Michigan Sediment at varying annealing temperatures Genus-level annotations are shown (when available), and the mean relative abundance (six technical replicates) for each primer set is shown, together with the difference in mean proportions. For each comparison a *q*-value, calculated in the software package STAMP using White’s non-parametric *t*-test along with a Benjamini–Hochberg FDR correction, is shown. Only significantly differently abundant taxa (*q* < 0.05) are shown. Sequences annotated as Stramenopiles are derived from SSU rRNA genes of chloroplasts from these organisms.

10.7717/peerj.6570/supp-4 Figure S4 Significant differences in taxon relative abundance between EMP and ShortEMP analyses for LMC, Feces, Soil and Zymo samples Genus-level annotations (except Zymo) are shown (when available), and the mean relative abundance (six technical replicates) for each primer set is shown, together with the difference in mean proportions. For each comparison a *q*-value, calculated in the software package STAMP using White’s non-parametric *t*-test along with a Benjamini-Hochberg FDR correction, is shown. Only significantly differently abundant taxa (*q* < 0.05) are shown. Zymo sequences were annotated to the taxonomic level of species by mapping to known references.

10.7717/peerj.6570/supp-5 Table S1 Original (EMP) and modified (ShortEMP) primer variants for 515F and 806R primers

10.7717/peerj.6570/supp-6 Table S2 Primer sequences used in this study

10.7717/peerj.6570/supp-7 Table S3 Mapping file metadata associated with all samples used in this study

10.7717/peerj.6570/supp-8 Table S4 Nucleotide diversity calculations for EMP and ShortEMP 515F primers

10.7717/peerj.6570/supp-9 Table S5 Nucleotide diversity calculations for EMP and ShortEMP 806R primers

## Additional Information and Declarations

### Data Availability

The following information was supplied regarding data availability: The raw sequence data files are available in the Sequence Read Archive (SRA) of the National Center for Biotechnology Information (NCBI) under PRJNA492144.
