# Supplementary figures and images for: PCR effects of melting temperature adjustment of individual primers in degenerate primer pools
Source: PeerJ. 2019 Mar 4;7:e6570. doi: 10.7717/peerj.6570 (PMC6404654; doi:10.7717/peerj.6570)

# Bray-Curtis Dissimilarity

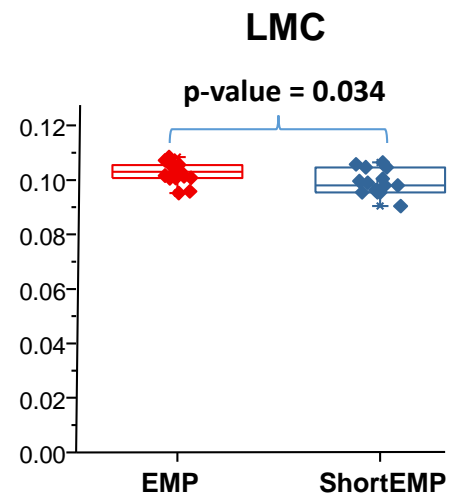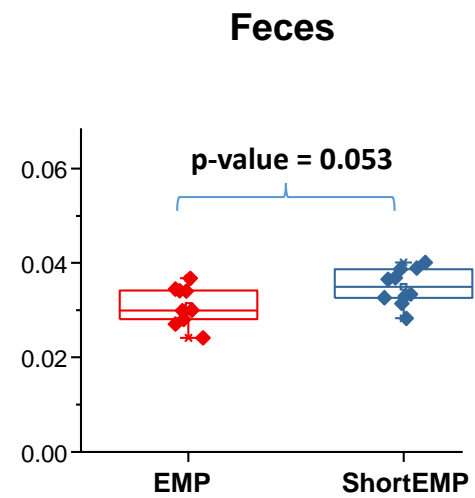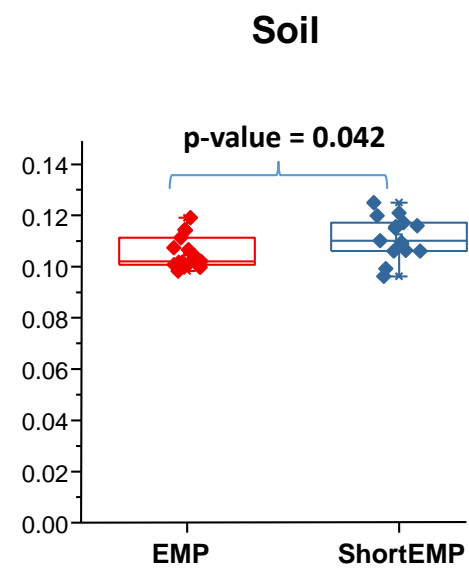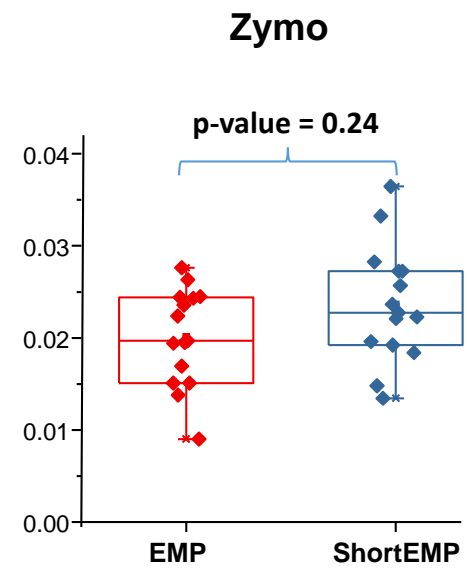

Supplement: Figure S1 — For each sample, Bray–Curtis dissimilarity was calculated for 6 technical replicates with EMP primers (15 comparisons), and 6 technical replicates with ShortEMP primers (15 comparisons). A comparison of median within-sample similarity for replicates from EMP and ShortEMP amplifications was performed, and were significantly different for LMC, Feces and Soil (Mann-Whitney test, P < 0.053). An outlier of one replicate from both EMP and ShortEMP fecal analyses was removed (see Fig. S2). [file peerj-07-6570-s001.pdf]

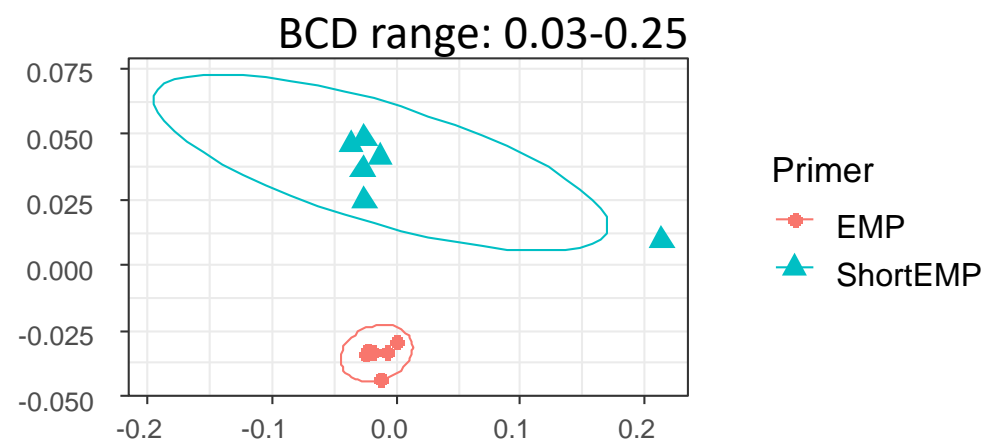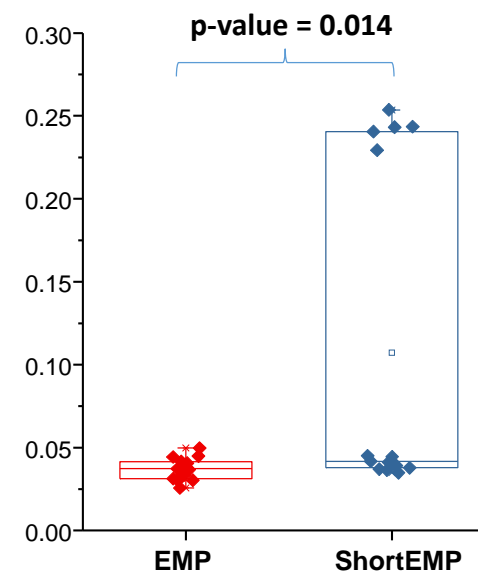

Supplement: Figure S2 — A single technical replicate, representing an outlier, was removed from both EMP and ShortEMP analyses (Fig. 3 and Fig. S1). Analyses in this figure are shown including the deep outliers. Inclusion of the outliers does not modify the conclusions of the analysis. However, the ShortEMP outlier is greatly different from all other technical replicates from all samples in the study. (A) Genus-level annotations of sequence data were visualized using mMDS ordination employing a distance matrix based on Bray–Curtis similarity. Six technical replicates were performed at optimal annealing temperatures of 45 °C (ShortEMP) and 50 °C (EMP). Small, but significant, shifts in microbial communities were observed between EMP and ShortEMP primers for Feces (ANOSIM R = 0.68, P = 0.0025). Bray–Curtis dissimilarity (BCD) between EMP and ShortEMP technical replicates is shown above the figure. Ellipses represent a 95% confidence interval around the centroid. (B) Box plot of within-group Bray–Curtis dissimilarity scores for microbiome analyses conducted with EMP and ShortEMP primer sets on fecal DNA. [file peerj-07-6570-s002.pdf]

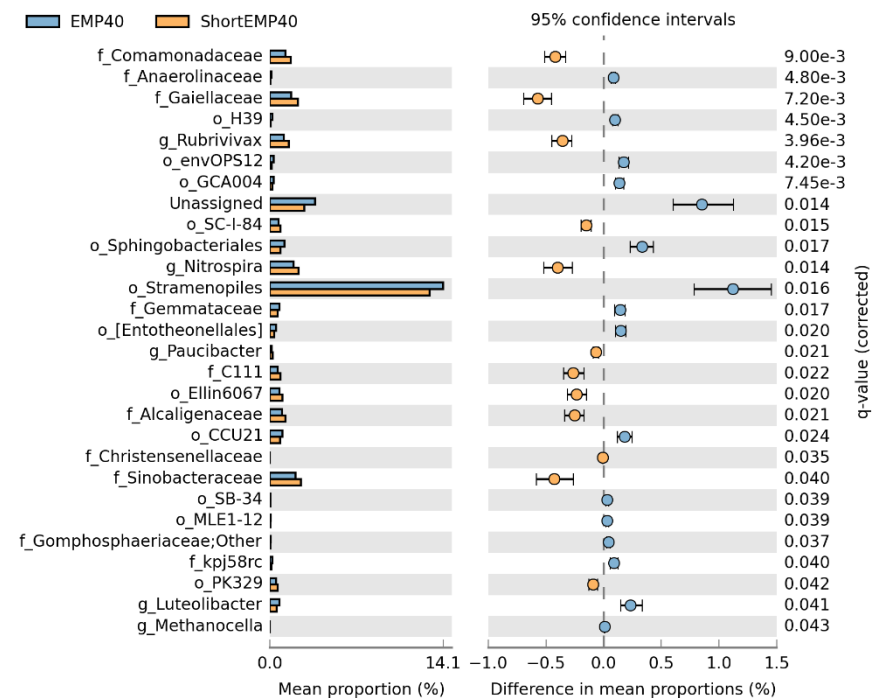

**40°C Annealing  
Temperature**

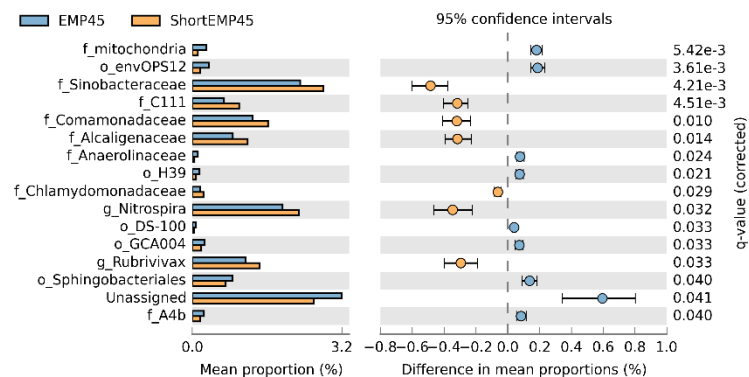

**45°C Annealing  
Temperature**

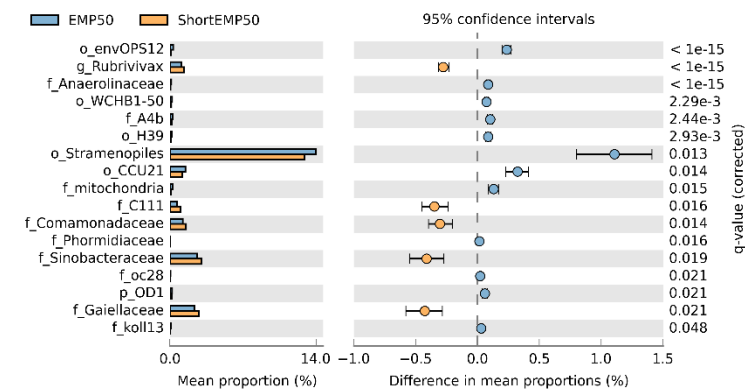

**50°C Annealing  
Temperature**

Supplement: Figure S3 — Genus-level annotations are shown (when available), and the mean relative abundance (six technical replicates) for each primer set is shown, together with the difference in mean proportions. For each comparison a q-value, calculated in the software package STAMP using White’s non-parametric t-test along with a Benjamini–Hochberg FDR correction, is shown. Only significantly differently abundant taxa (q < 0.05) are shown. Sequences annotated as Stramenopiles are derived from SSU rRNA genes of chloroplasts from these organisms. [file peerj-07-6570-s003.pdf]

**(A) Lake Michigan Sediment**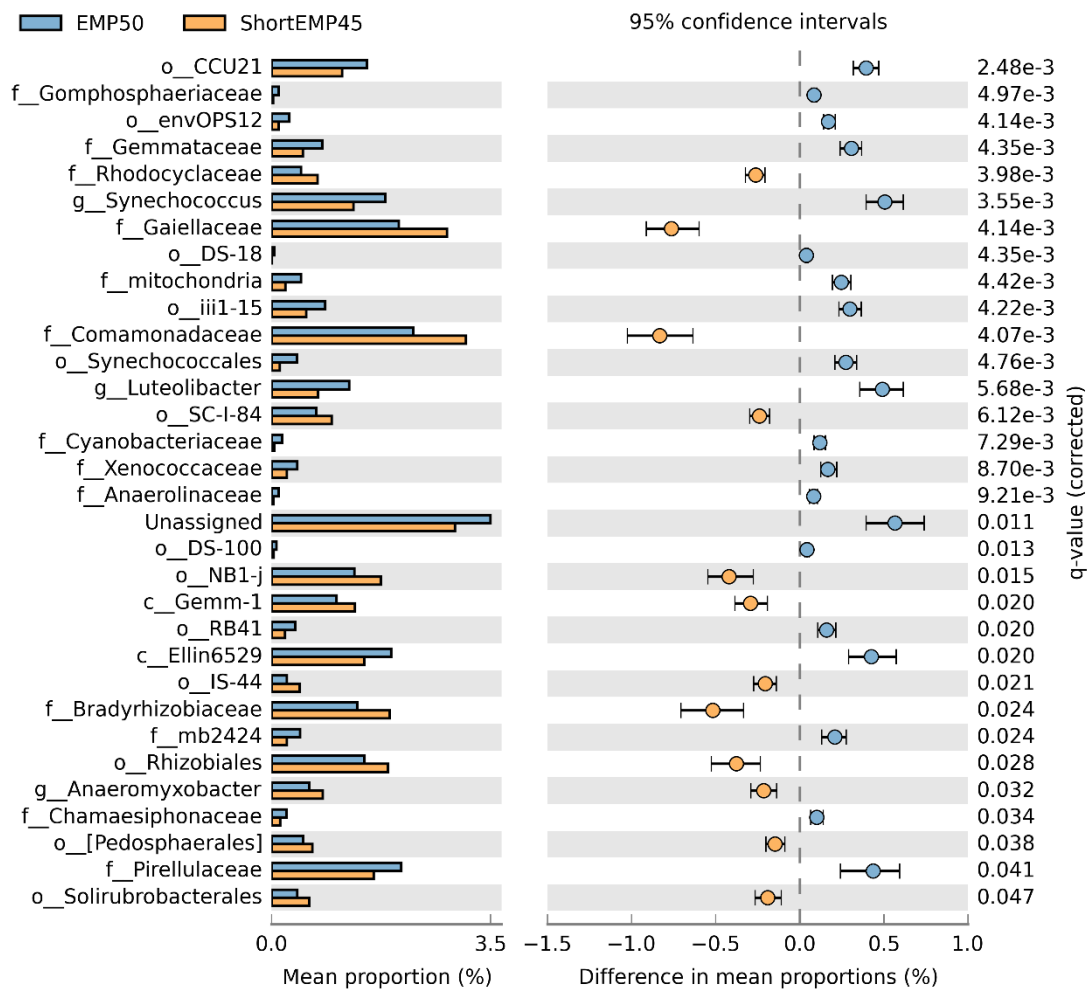**(B) Feces**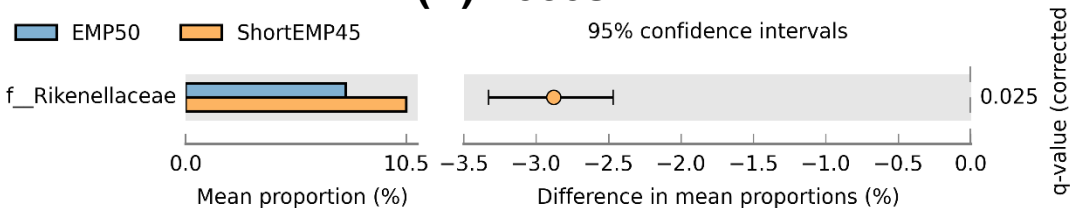**(C) Soil**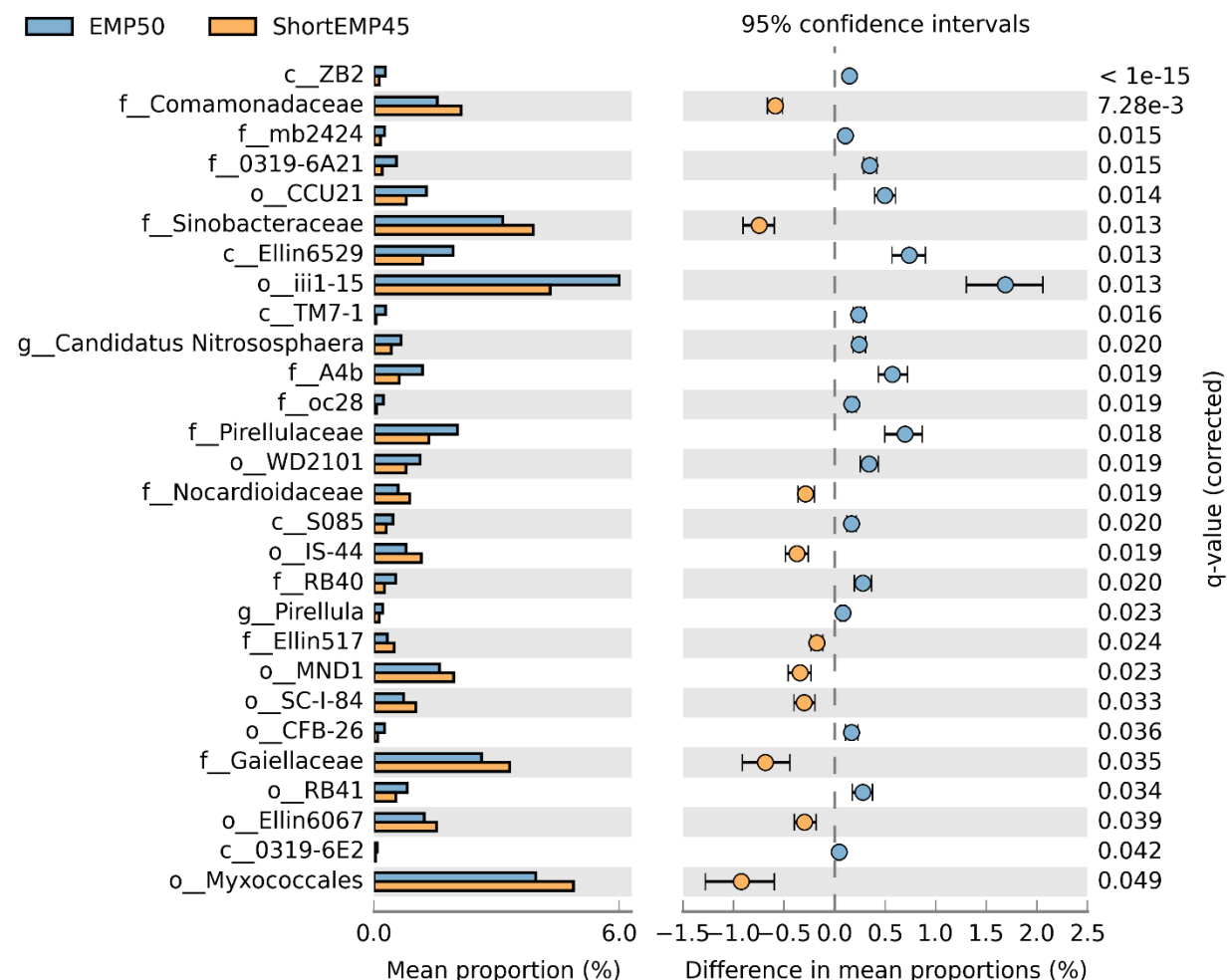**(D) Zymo Standard**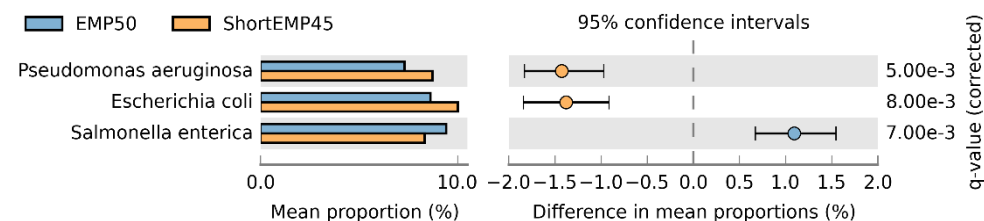

Supplement: Figure S4 — Genus-level annotations (except Zymo) are shown (when available), and the mean relative abundance (six technical replicates) for each primer set is shown, together with the difference in mean proportions. For each comparison a q-value, calculated in the software package STAMP using White’s non-parametric t-test along with a Benjamini-Hochberg FDR correction, is shown. Only significantly differently abundant taxa (q < 0.05) are shown. Zymo sequences were annotated to the taxonomic level of species by mapping to known references. [file peerj-07-6570-s004.pdf]
